# Protein Biochemistry and Expression Regulation of Cadmium/Zinc Pumping ATPases in the Hyperaccumulator Plants *Arabidopsis halleri* and *Noccaea caerulescens*

**DOI:** 10.3389/fpls.2017.00835

**Published:** 2017-05-22

**Authors:** Seema Mishra, Archana Mishra, Hendrik Küpper

**Affiliations:** ^1^Fachbereich Biologie, Mathematisch-Naturwissenschaftliche, Universität KonstanzKonstanz, Germany; ^2^Department of Biophysics and Biochemistry of Plants, Institute of Plant Molecular Biology, Biology Centre of the ASCRČeské Budějovice, Czechia; ^3^CSIR-National Botanical Research Institute, Plant Ecology and Environmental Science DivisionLucknow, India; ^4^Department of Experimental Plant Biology, Faculty of Science, University of South BohemiaČeské Budějovice, Czechia

**Keywords:** cadmium, heavy metal ATPase, metal hyperaccumulator plants, QISH, RT-qPCR, zinc

## Abstract

P_1B_-ATPases are decisive for metal accumulation phenotypes, but mechanisms of their regulation are only partially understood. Here, we studied the Cd/Zn transporting ATPases NcHMA3 and NcHMA4 from *Noccaea caerulescens* as well as AhHMA3 and AhHMA4 from *Arabidopsis halleri*. Protein biochemistry was analyzed on HMA4 purified from roots of *N. caerulescens* in active state. Metal titration of NcHMA4 protein with an electrochromic dye as charge indicator suggested that HMA4 reaches maximal ATPase activity when all internal high-affinity Cd^2+^ binding sites are occupied. Although HMA4 was reported to be mainly responsible for xylem loading of heavy metals for root to shoot transport, the current study revealed high expression of NcHMA4 in shoots as well. Further, there were additional 20 and 40 kD fragments at replete Zn^2+^ and toxic Cd^2+^, but not at deficient Zn^2+^ concentrations. Altogether, the protein level expression analysis suggested a more multifunctional role of NcHMA4 than previously assumed. Organ-level transcription analysis through quantitative PCR of mRNA in *N. caerulescens* and *A. halleri* confirmed the strong shoot expression of both *NcHMA4* and *AhHMA4*. Further, in shoots *NcHMA4* was more abundant in 10 μM Zn^2+^ and *AhHMA4* in Zn^2+^ deficiency. In roots, *NcHMA4* was up-regulated in response to deficient Zn^2+^ when compared to replete Zn^2+^ and toxic Cd^2+^ treatment. In both species, *HMA3* was much more expressed in shoots than in roots, and *HMA3* transcript levels remained rather constant regardless of Zn^2+^ supply, but were up-regulated by 10 μM Cd^2+^. Analysis of cellular expression by quantitative mRNA *in situ* hybridisation showed that in *A. halleri*, both *HMA3* and *HMA4* mRNA levels were highest in the mesophyll, while in *N. caerulescens* they were highest in the bundle sheath of the vein. This is likely related to the different final storage sites for hyperaccumulated metals in both species: epidermis in *N. caerulescens*, mesophyll in *A. halleri*.

## Introduction

Hyperaccumulator plants actively take up metals or metalloids and store them in above ground parts particularly in the vacuoles of large epidermal cells. In this way, these plants can tolerate and accumulate high amounts, up to several percent of shoot dry biomass, of metal/metalloid. The ability of hyperaccumulators can be exploited for phytomining, the commercial extraction of metals from soils (Baker and Brooks, 1989; Chaney et al., 2005) and for phytoremediation purposes (Chaney, 1983; Küpper and Kroneck, 2005). Strongly enhanced xylem loading of the metal ion in the root, increased root-to-shoot transport and highly efficient metal detoxification are the common attributes of metal hyperaccumulation (Lasat et al., 1996; Küpper et al., 1999; Krämer et al., 2000). It involves active pumping of the metals into specific storage sites, such as vacuoles of the epidermal cells, where the concentrations of heavy metals can reach up to several hundred mmol.l^-1^ (Küpper et al., 1999, 2001). It has been concluded that transport steps over the plasma and tonoplast membranes of leaf epidermal storage cells are the main driving forces leading to the hyperaccumulation phenotype (Leitenmaier and Küpper, 2011). Several families of transporters, like P_1B_-type ATPases, ZIPs, Nramps, CDFs, and CAXs are involved in uptake, translocation, and vacuolar sequestration of metals (Hall and Williams, 2003; Küpper and Kroneck, 2005; Williams and Mills, 2005). In hyperaccumulators many of these transporters are naturally overexpressed. Therefore, apart from above mentioned applications, hyperaccumulator plants are also excellent models for gaining further understanding of general metal transport mechanisms. As the main metal transporter protein families are common in all eukaryotes and even in prokaryotes (such as P_1B_ type ATPases, Williams and Mills, 2005), the mechanistic insights would be applicable to other organisms as well, including humans. The P_1B_-type ATPases, a subfamily of the P-type ATPases, are ubiquitous transmembrane proteins that use the hydrolysis of ATP to ADP and phosphate as a driving force to pump metal ions across membranes against an electrochemical gradient. They are found in all kingdoms, including humans (Solioz and Vulpe, 1996). Malfunction of P_1B_-type ATPases results in several lethal diseases in humans (Bull and Cox, 1994). Among P_1B_-type heavy metal transporting ATPasas, the Heavy Metal Associated 4 (HMA4) protein has been studied most. It is mainly localized in the plasma membrane of the pericycle cell layer of the root vasculature (Verret et al., 2004; Sinclair et al., 2007). HMA4 is involved in xylem loading of Cd and Zn (Papoyan and Kochian, 2004; Verret et al., 2004), and therefore plays key role in root to shoot translocation of Zn and Cd (Mills et al., 2005; Wong and Cobbett, 2009). On the other hand, HMA3, located in the vacuolar membrane (Morel et al., 2009; Miyadate et al., 2011), participates in vacuolar sequestration of Zn, Cd, cobalt (Co), and lead (Pb). Much higher expressions of genes for both HMA4 and HMA3, compared to related non-tolerant, non-accumulator species, were found in the Cd/Zn hyperaccumulators *Noccaea caerulescens* (formerly *Thlaspi caerulescens*) (Papoyan and Kochian, 2004; Van de Mortel et al., 2006; Ueno et al., 2011) and *Arabidopsis halleri* (Becher et al., 2004; Weber et al., 2004). Recently Zhang et al. (2016) reported an overexpression of HMA2 instead of HMA4 in roots of Cd/Zn hyperaccumulator *Sedum alfredii*. In non-hyperaccumulators, such as *Arabidopsis thaliana* and rice, expression of HMA2 or HMA3 leads to decreased Cd and Zn content in the shoots (Eren and Argüello, 2004; Gravot et al., 2004; Tezuka et al., 2010; Ueno et al., 2010; Miyadate et al., 2011). Since hyperaccumulators are well-known for having an enhanced root to shoot translocation, the enhanced expression of HMA transporters indicates their different roles in hyperaccumulators compared to non-accumulators.

While the general over-expression of metal transporters in hyperaccumulators is well known on the whole-plant and tissue level, expression and its metal-dependent regulation on a single-cell level is less investigated. By using a quantitative mRNA *in situ* hybridisation (QISH) technique, important mechanistic information about regulation of Zn transport has been obtained in *N. caerulescens* (Küpper et al., 2007b; Küpper and Kochian, 2010). The expression analysis through QISH revealed that in *N. caerulescens*, the Zn-transporter ZNT1, highly expressed in the photosynthetic cells, have no role in Zn hyperaccumulation (Küpper et al., 2007b). Instead ZNT5, possibly together with MTP1 (=ZTP1), is a key player in the sequestration of Cd and Zn into the epidermal storage cells (Küpper and Kochian, 2010).

The crystal structures of two bacterial P_1B_ ATPases for copper (Gourdon et al., 2011) and zinc (Wang et al., 2014) transport have been published in the recent years, but the crystal structure of a eukaryotic full-length heavy metal ATPase is still completely lacking. The first crystal structure indicated a three-stage pathway of metal transport, in which conserved residues play a decisive role and the step requiring ATP hydrolysis is the release of the copper (Gourdon et al., 2011). The second structure further clarified the importance of two conserved cysteine residues in the high affinity metal binding site, and via comparison of two crystallized states of the enzymatic cycle it provided better insight into the pumping mechanism (Wang et al., 2014).

Such crystal structures cannot reveal, however, all the biochemical characteristics and features that are required for the enzymatic function of these proteins, such as in which metal concentration range they are really active, what are the activation energies, *etcetera*. Only few studies have reported biochemical and biophysical characterisations of such enzymes, either in bacteria or plants or animals including humans (Hung et al., 2007; Parameswaran et al., 2007; Liu et al., 2010; Leitenmaier et al., 2011). The earlier two of these studies paved the way for purifying such proteins in active state, and Hung et al. (2007) already showed the copper concentration range where ATP7A (Menkes’ Protein) becomes activated and inhibited, with an EC50 value of 0.7 μM for Cu(I)-dependent ATPase activity. Similar values were found for Cd^2+^, Cu^2+^, and Zn^2+^ in the Cd/Zn-ATPase NcHMA4 (=TcHMA4) by Leitenmaier et al. (2011), who furthermore determined the activation energies of the ATPase activity with these metals and their dependence on the metal concentration. Despite the two crystal structures and the biochemical–biophysical studies several questions still remain open, such as details of the pumping mechanism. The main reason for dearth of such studies on the level of isolated proteins is firstly, the membrane proteins are difficult to over-express either homologously or heterologously with correct folding and secondly, they are relatively short lived in terms of activity due to the extreme abundance of cysteine residues (58 in the mRNA sequence) which easily gets oxidized after purification (Leitenmaier et al., 2011). In the current study, the metal binding aspects of HMA4, purified from *N. caerulescens*, was analyzed in active state. Further, the expression regulation of both HMA3 and HMA4, reported to exhibit different functional roles in heavy metal transport, was analyzed at tissue and cellular level in Cd/Zn hyperaccumulators *A. halleri* and *N. caerulescens.* Since the two hyperaccumulators have contrasting metal storage mechanisms, the comparative study would reveal new aspects of the molecular biological mechanisms leading to metal hyperaccumulation.

## Materials and Methods

### Plant Material and Culture Conditions

The Ganges ecotype of *Noccaea caerulescens* (J. Presl and C. Presl) F. K. Mey. (formerly called *Thlaspi caerulescens* J. Presl and C. Presl) and *Arabidopsis halleri* (Linné) O’ Kane and Al-Shehbaz (formerly called *Cardaminopsis halleri* L.) were grown hydroponically in a controlled environment chamber. The seeds for the current experiments were from seed increases in the lab of H. Küpper, but seeds for the first generation of both species in this lab were provided in 1999 by the lab of S. McGrath (Rothamsted Experimental station, United Kingdom) who collected them in the field. The “Ganges” ecotype of *N. caerulescens* was originally referred to as “French A” and originates from a Zn/Pb mining site in the Cevennes region (Lombi et al., 2000). For germination, the seeds were spread on moistened perlite: vermiculite (3:1) and incubated at 4°C for 1 week, then germinated at 20–25°C. The 3-week-old seedlings were transferred on plastic pots filled with nutrient solution (Küpper et al., 2007a). The nutrient solution was aerated continuously using a lab built system and automatically renewed by a programmable peristaltic pump (Ismatec MCP process). The nutrient medium contained either 0, 10, 100 μM Zn or 10 μM Cd along with 10 μM Zn. The growth chamber was maintained at 14 h day length and 22°C (day)/18°C (night) temperature. The photon flux density during the light period followed an approximately sinusoidal cycle with a maximum around 150 μmol⋅m^-2^⋅s^-1^ and was supplied by full-spectrum discharge lamps.

### Isolation and Purification of HMA4

#### Chemicals

Loading of the protein with metals present in regular analytical grade chemicals caused problems in further characterization of HMA4. Therefore, most of the chemicals used for isolation and all the chemicals used for buffers after dialysis were used in highest grade available (Suprapur^®^ or Ultrol^®^) purchased from Merck (Darmstadt, Germany) except for the following. Mannitol was purchased from Sigma-Aldrich (St. Louis, MO, United States), in the highest grade available (SigmaUltra), the detergent *n*-dodecyl-β-maltoside (DDM) and the antioxidant tris(2-carboxyethyl)phosphine (TCEP) from Hampton Research (Aliso Viejo, CA, United States), the protease inhibitor “Protease Inhibitor Cocktail tablets complete EDTA-free” (Roche Diagnostics, Mannheim, Germany), ATP magnesium salt from Merck (Darmstadt, Germany) as “Low metals Grade,” the metal chelator “Chelex Resin 100” from Bio-Rad (Hercules, CA, United States).

#### Column and Other Materials

A “Superformance SC” (Götec-Labortechnik GmbH, Bickenbach, Germany) with 26 mm inner diameter and variable length was packed with “Protino” Ni-IDA, Macherey-Nagel, (Düren, Germany) IMAC resin to about 30 ml bed volume. The column was cooled to about 2°C with its thermostat jacket. Other pre-column and post-column materials used were: wheat mill: Jupiter 872 with Messerschmidt ceramic grinding engine (Messerschmidt Hausgeräte GmbH, Königsfeld-Erdmannsweiler, Germany), ultracentrifuge: Beckmann LE-80 (Beckmann, Palo Alto, CA, United States), AAS: GBC 932 AA (Dandenong, VIC, Australia), centrifugal protein concentrators: Amicon Ultra-15, membrane with 10 kD exclusion size (Millipore Corporation, Bedford, MA, United States).

#### Isolation of Protein

Isolation and purification of the protein was based on the method developed by Parameswaran et al. (2007) and Leitenmaier et al. (2011), with anoxic conditions described herein, in detail. The buffers used from protein solubilization onward (given below), including for purification and characterization, were made anoxic by flushing with nitrogen. First, the vacuum was applied for 10 min to pull out the dissolved air and then aerated with nitrogen for 10 min, this cycle was repeated seven times. The anoxic buffers were placed in an anoxic glove box until used. The chemicals to be added right before use were added in glove box and pH was adjusted if needed.

The roots of *N. caerulescens* exposed to 100 μM Zn were used for the isolation of HMA4 by the method described earlier (Leitenmaier et al., 2011) with slight modifications. Briefly, the isolation buffer contained 330 mM mannitol, 30 mM HEPES, 3 mM MgCl_2_, 2% PVP, and 10 mM TCEP. The pH was adjusted to 6.0 at 4°C with KOH and “complete” EDTA-free protease inhibitor (1 tablet/50 ml buffer) was added freshly. The roots were ground with frozen droplets of isolation buffer in 1:5 (w/v) ratio to fine powder, thawed under nitrogen and centrifuged twice at 246,000 × *g* for 1 h at 4°C. After each run the supernatant was discarded and the pellet was re-dissolved in isolation buffer. Finally, the pellet was transferred to solubilisation buffer (1:2, w/v) anoxically and solubilised overnight at 4°C with continuous stirring at 250 rpm. The composition of solubilisation buffer was; 160 mM NaH_2_PO_4_ (Merck suprapur grade), 1.6 M NaCl. 10 mM TCEP and 10 mM DDM was dissolved, pH was adjusted to 6.0 at 4°C with KOH, then “complete” was added as mentioned above. From this step the protein was handled only in the anoxic glove box. The solubilised protein was centrifuged at 246,000 × *g* for 1 h at 4°C and the supernatant was collected in tightly closed bottles and stored on ice.

#### Purification of Protein

All the buffers were prepared a day before and cooled, the desired pH were adjusted at 4°C and then anoxicated and stored and handled in glove box.

The solubilized protein was diluted to 240 ml with buffer A (0.5 mM NaH_2_PO_4_, 49.5 mM HEPES and 300 mM NaCl, pH 9.0, 0.2 mM DDM added freshly) and final pH was adjusted to 9.0 for efficient binding to the column. An aliquot of protein before and after dilution was secured to confirm integrity of the protein via SDS gels and Western blots. The diluted protein was centrifuged for an hour and loaded to the column, which was cooled to 2°C.

Under present (anoxic) conditions the binding efficiency of protein to the column was greatly enhanced compared to the previous oxic conditions, and complete elution of protein from Ni column was difficult. Therefore, subsequently the column was regenerated with Zn^2+^ to increase the specificity as NcHMA4 is a Cd/Zn-pumping ATPase, which was not possible in oxic conditions where HMA4 did not sufficiently bind to Zn^2+^ loaded columns (Parameswaran et al., 2007). The Zn^2+^ column gave sufficient binding and improved the purity of protein.

After loading, the column was washed with buffer A until a stable baseline was attained and HMA4 was eluted with a gradient of buffer A and buffer B (0.5 mM NaH_2_PO_4_, 49.5 mM HEPES, 300 mM NaCl and 1 M imidazole, pH 6.0, 0.2 mM DDM added freshly) with B reaching to 100% in 1 h 20 min, stayed at 100% until 2 h (end of elution). The eluted fractions were concentrated through 10 kD cut off concentrators (Amicon Ultra-15, Millipore Corporation, Bedford, MA, United States). The fractions of interest, checked on a drop blot (Western blot), were dialyzed using floating re-usable micro dialysis capsules (QuixSep^®^, Carl Roth, Karlsruhe, Germany) against the buffer containing 0.5 mM NaH_2_PO_4_, 49.5 mM HEPES and 300 mM NaCl, pH 6.0 and 10% Chelex to obtain metal free protein for further characterization. The buffer used for dialysis was filtered to remove Chelex and used (as metal free buffer) during characterization of protein.

### Methods for Testing Protein Identity and Purity

The purity of NcHMA4 was checked via SDS-Page and Western blots as described in earlier (Leitenmaier et al., 2011). In brief, the concentrated protein fractions were precipitated with 20% TCA in duplicate and the pellets were dissolved in sample buffer (0.125 M Tris pH 6.8 with HCl, 20% (w/v) Glycerol, 4% (w/v) SDS, 10% (w/v) DTT, 0.002%-0.004% (w/v) bromphenol blue). Two identical gels were loaded and run in parallel and after the run, one gel was used for Western blotting with an antibody specific for a loop region of HMA4 as described in Parameswaran et al. (2007) whilst the other gel was either stained through silver staining (Parameswaran et al., 2007) or with Lumitein Protein Stain (Biotium, Hayward, CA, United States). Visualization of Lumitein staining was achieved using the LumiImager and the LumiCapt Software (Boehringer, Mannheim, Germany).

### Methods for Quantification of Protein

Known concentrations of BSA standards were prepared in the same way along with samples, as mentioned above, and loaded onto the SDS gel. After the run, the gel was stained with Lumitein Protein Stain (Biotium, Hayward, CA, United States). Quantification was accomplished using the LumiImager and the LumiCapt Software (Boehringer, Mannheim, Germany).

### ATPase Activity Assay

To verify if the purified protein is active and usable for further characterization, the ATPase activity assay was performed with various concentrations of Cd and Zn by the method optimized by Leitenmaier et al. (2011) by following the release of phosphate. Standards of phosphate (0 to 1200 μM) were used for the standard curve.

### Measurement of Metal Binding to the Protein through Charge Transfer Spectra

The cuvette (150 μl) was equilibrated with the buffer and filled with 100 μl diluted protein (OD_280_ = 0.11; about 0.6–0.7 μg/ml final concentration) in the buffer. The spectrum was recorded from 250 to 800 nm on the UV/VIS spectrophotometer Lambda 750 (Perkin-Elmer, Germany) at a spectral bandwidth of 1 nm with a 0.2 nm recording interval. The protein was titrated with Cd (0.1 to 200 μM Cd final concentration). The spectra of metal titration were normalized between 0 and 1 and were subtracted by the spectrum of only protein by using Microcal Origin Professional 8.6. The increase in absorption at 375 nm due to charge transfer from ligand to metal was observed.

### Charge Movement in the Membrane through Electrochromic Fluorescent Dye

Lipid vesicles were prepared from phosphatidylcholine from soybean (type II-S, from Sigma-Aldrich) that was sonicated at a concentration of 66 μg/ml in metal-free buffer A. Fluorescence experiments were carried out in a Perkin Elmer LS50B fluorescence spectrophotometer. The cuvette holder was equipped with a magnetic stirrer. The excitation wavelength was set to 480 nm and the emission wavelength to 550 nm. The F5 dye concentration of 200 μM in the working stock was diluted from a storage stock solution of 20 mM in ethanol.

The cuvette with stirring bar, made of quartz [type 109.004F-QS from Hellma, Müllheim (Baden), Germany], was equilibrated with dye by filling with 1 ml buffer containing 1 μl (0.2 μM final concentration) dye. The pre-equilibrated cuvette was then filled with buffer containing lipid (6.6 μg/ml) and placed in the cuvette holder with constant stirring. After a stable base line was achieved, the following was added sequentially after achieving stable fluorescence signal at each step: 0.2 μM dye, 150 μl (0.3 μg/ml) protein, ATP 5 mM, 1 μM Cd or Mg (final concentrations in 1 ml). Fluorescence changes, caused by additions of reagents, were determined as relative signal changes with respect to the fluorescence level after the addition of dye. The experiments were carried out in climatic controlled room at 20°C.

### Expression of NcHMA4 – Protein Level

The expression of NcHMA4 protein was analyzed in 2–3 months old plants grown in hydroponic system as described above. The expression was checked via SDS-Page and Western blots. The NcHMA4 was isolated in the buffer containing; 750 mM aminocaproic acid, 50 mM Bis-Tris and 2% PVP, after adjusting the pH to 7.6 10 mM TCEP was added. At last “complete” EDTA-free protease inhibitor (1 tablet/50 ml buffer) was added freshly. The root and shoot samples 250–500 mg were frozen and ground to fine powder in liquid nitrogen along with frozen droplets of isolation buffer in 1:2 ratio (w/v). The samples were centrifuged at 246,000 × *g* for 1 h at 4°C and supernatant was discarded and pellet was re-dissolved in isolation buffer. The pellet was washed twice in this way and dissolved in solubilisation buffer (same as isolation buffer plus 10 mM DDM). The protein was solubilized overnight at 4°C with slow continuous stirring. The solubilized protein samples were precipitated with 20% TCA and gel running and Western blotting were performed by the method of Parameswaran et al. (2007) as mentioned above.

### Tissue-Level *HMA* Expression Analysis: RNA Extraction and Quantitative qRT-PCR

Total RNA from roots and shoots of 2–3 months old *N. caerulescens* and *A. halleri* plants was isolated with TRIzol reagent (Ambion Technologies, United States) and digested with DNase I (RQ1 RNase-Free DNase; Promega, United States) according to the manufacturer’s instructions. RNA integrity (RIN > 8) was confirmed using a Nano drop 100 photometer (Thermo Fisher Scientific, United States).

After treatment with DNaseI, cDNA was synthesized using the Maxima First Strand cDNA Synthesis Kit for RT-qPCR (Cat. No.: 1641; Biogen) according to the supplier’s instructions using 1 μg RNA per reaction. PCR was carried out with a Bio Rad CFX96 (Bio-Rad) in a total volume of 20 μl including SYBR Premix Ex Taq (Tli RNaseH Plus) (Takara Bio, United States), 0.3 μM (reference gene) and 0.6 μM of each gene-specific primer (**Table 1**). Cycling conditions were as follows: initial treatment (3 min, 95°C), denaturation (10 s, 95°C), annealing (30 s, 56°C), extension (15 s, 60°C) for 40 cycles. The reaction was stopped by 30 s incubation at 95°C. Product specificity was tested by melting curve analyses (65–95°C). PCR efficiencies were checked in all cases and were between 90 and 115%. Two reference genes were used for normalization: cytosolic glyceraldehyde 3-phosphate dehydrogenase (c*GAPDH*, EC 1.2.1.12) and 18S ribosomal RNA (*18S rRNA*) (**Table 1**). Data collection was performed from two independent experiments with at least three different plants measured per experiment. To calculate the transcript abundance and standard error (SE) for the target group and the control group, the method Pfaffl (2001) was used.

**Table 1:** Primers used in real time quantitative RT-PCR analysis in the present study.

Sl. No.	Gene	Description	Primer sequence (5′–3′)
1	cGAPDH	Glyceraldehyde 3-phosphate dehydrogenase (cytosolic)	F-TGCACCACTAACTGCCTTGC R-AAGCACCTTTCCGACAGCCT
2	18sRNA	18S Ribosomal RNA	F-CTGGCGACGCATCATTCAAA R-CTGCCTTCCTTGGATGTGGT
3	AhHMA3	*Arabidopsis halleri* Heavy metal ATPase 3	F-GCCTAAGCCTGATCTCGTTG R-TCCGGAACTGTTAAGCATCC
4	AhHMA4	*Arabidopsis halleri* Heavy metal ATPase 4	F-CTGCAGCGATGAAAAACAAAC R-TAAGGCTTCTCACCGCAGATT
5	NcHMA3	*Noccaea caerulescens* Heavy metal ATPase 3	F-ATCAACCTCTGTCGAGCCTAAG R-TCCTTCTCCTGGAAAGTTCTGA
6	NcHMA4	*Noccaea caerulescens* Heavy metal ATPase 4	F-CAACTTGTATGGTAGGAGATGGT R-GCTGAGCTCTTCTTGCTAGCTTT

### Cellular Expression Regulation of *HMA3* and *HMA4* from *N. caerulescens* and *A. halleri*

The analysis of cellular localization of gene regulation was done by the QISH method developed by Küpper et al. (2007b). Briefly, the young-mature leaves of 2- to 3-month-old *N. caerulescens* and *A. halleri* were cut into 2 mm × 2 mm pieces. The samples were then fixed and dehydrated for pigment extraction. Pigment extraction was performed to remove auto-fluorescence background, remaining traces of chlorophylls and their degradation products were made non-fluorescent by conversion to the respective copper complexes. After re-hydration and protein digestion, the samples were hybridized with the fluorescence labeled probes of *NcHMA3* and *NcHMA4* and *AhHMA3* and *AhHMA4*, respectively. The probes (fluorescently labeled DNA oligonucleotides given in **Table 2**) were designed to have identical melting temperatures and GC content and no problematic features like self-dimerization. The fluorescence label Cy5 was used for the gene of interest and Bodipy-TMR-X for the internal standard (cGAPDH), for the reasons described in detail by Küpper et al. (2007b). For QISH image processing ImageJ software was used. Four independent experiments, each with at least three plants, were analyzed by QISH.

**Table 2:** Probes used in QISH in the present study.

Sl. No.	Gene	Description	QISH probe sequence (5′–3′)
1	anti-cGAPDH	Glyceraldehyde 3-phosphate dehydrogenase (cytosolic)	ACCCTTCAAGTGAGCAGCAGCCTTGTCCTTGTCA
2	anti-AhHMA3	*Arabidopsis halleri* Heavy metal ATPase 3	CTCTGCTTCATCCTCCTCAAGTTTCACCGGCGAA
3	anti-AhHMA4	*Arabidopsis halleri* Heavy metal ATPase 4	CACAGAGGGGGAAGTGGGAGAACAAACTGATCTG
4	anti-NcHMA3	*Noccaea caerulescens* Heavy metal ATPase 3	AGGAAGGAGAGAGCAAGCAACACACCAGAAGCCA
5	anti-NcHMA4	*Noccaea caerulescens* Heavy metalATPase4	CACCGCTCTCCACCACCACATTGACCAAACTAGA
6	Non-binding	Non-binding sequences	GCATTAGCCACGGTAGCGATAGGTAGCATTGGAG

### Statistics

Comparison of groups for finding statistically significant differences was performed via two-way ANOVA’s in SigmaPlot (Version 11, SPSS Science, United States) at the significance level *P* < 0.05. In case of significant effects, the Holm–Sidak method was used for an all-pairwise multiple comparison. All other analysis of the data was done in Origin professional (versions 8.5 and 2015, OriginLab, United States).

## Results and Discussion

### ATPase Activity Analysis of Metal Binding to the Protein

The metal activation of the NcHMA4 ATPase activity with various concentrations of Cd and Zn (Leitenmaier et al., 2011) was measured also in the current work to verify the activity of protein for the further steps of characterization. The result confirmed the previous observation of activation optimum between 0.03 and 1 μM, i.e., submicromolar metal concentrations (Leitenmaier et al., 2011). On this basis, the detailed characterization of NcHMA4 with respect to metal binding properties and the movement of charge into the membrane was carried out.

**FIGURE 1:**
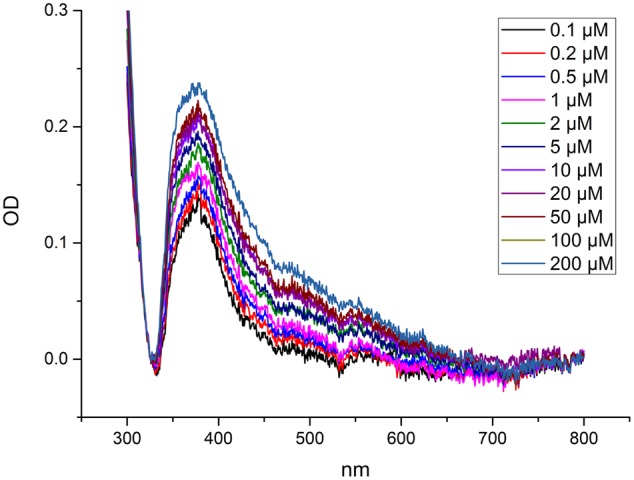
**Measurement of metal binding to NcHMA4 protein through ligand-metal charge transfer (LMCT) spectra.** Difference spectra showing absorption changes of NcHMA4 in response to the addition of increasing amounts of Cd^2+^. The 100 μl protein containing approximately 0.6–0.7 μg/ml protein was titrated by 0.1–200 μM Cd^2+^ final concentration. The figure is representative for two independent experiments.

Metal binding was analyzed via the change of a characteristic ligand to metal charge transfer (LMCT) band at 375 nm (**Figure 1**). The changes in the spectra were obtained by titrating the protein with Cd^2+^. The LMCT band at 375 nm was used to determine at which Cd^2+^ concentration (at large molar excess of Cd compared to the protein) NcHMA4 would be fully saturated with Cd^2+^, in comparison to its activation by Cd^2+^. This measurement yielded no clear saturation, but a continuous increase of the band up to 200 μM Cd was observed (**Figure 1**), which was far above the total inhibition of activity. Therefore, this method was not developed further, but the result obtained shows that this protein remains a target of Cd^2+^ binding also at highly toxic Cd^2+^ concentrations.

**FIGURE 2:**
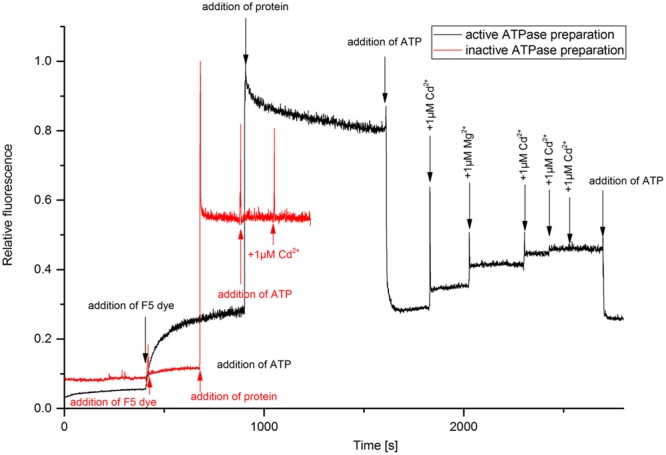
**Analysis of metal binding to NcHMA4 protein through the electrochromic fluorescent dye F5.** Fluorescence kinetic response of the dye inserted together with NcHMA4 into lipid vesicles to subsequent addition of 5 mM ATP and 1 μM Cd^2+^ per step. The final concentrations of dye and protein were 0.2 μM, 0.3 μg/ml, respectively. The figure is representative for three independent experiments.

The kinetic assay with the electrochromic fluorescence styryl dye F5 was used to study the enzyme-mediated charge translocation processes. The dye inserts in the membrane containing the enzyme and allows the optical recording of membrane potentials, i.e., the fluorescence of dye changes depending on charges in its vicinity as described in detail by Apell and Bersch (1987). The fluorescence response of the dye was monitored upon different substrate additions (**Figure 2**). The experiment enables the detection of electrogenic ion binding in the membrane, and release from the membrane (Bühler et al., 1991). It was more successful now than the titration of the LMCT band in yielding data regarding the Cd^2+^ binding capacity of HMA4. Addition of protein in the buffer containing dye caused a strong fluorescence increase due to the insertion of the dye molecules in the membrane. The partition coefficient of this type of dyes is very high (∼250,000), so that >90% of the dye molecules insert in the membrane (Bühler et al., 1991). Addition of ATP caused dropping of fluorescence when added to an assay containing active enzyme, but not when inactive enzyme was used in otherwise identical conditions. This excludes that the fluorescence change was due to a pH drop or ionic strength effect, both of which would be the same with inactive enzyme. The possible explanation for this fluorescence quenching is that probably the dye sitting with protein was surrounded with positive charge (due to lower pH 6.0 or presence of Na^+^ in the buffer) prior to addition of ATP. Upon addition of ATP (negative charge) the membrane potential neutralized. Further addition of Cd^2+^ and Mg^2+^ increased the fluorescence due to substrate binding – Mg^2+^ is known to activate most ATPases. The fluorescence quantum yield of F5 increased upon addition of Cd^2+^ only when the active form of NcHMA4 was reconstituted into artificial lipid vesicles, old or low-quality preparations of NcHMA4 as judged by the ATPase activity test did not work in this assay (**Figure 2**). The sequential addition of Cd^2+^ showed that the binding capacity slowly started to saturate and completely saturated at 3 μM Cd^2+^, which was the Cd^2+^ concentration when NcHMA4 also exhibited maximal activity. Addition of ATP at this stage further neutralized the charge causing fluorescence quenching. However, no further binding of Cd^2+^ occurred, possibly due to protein inactivation. Since the very hydrophobic F5 dye is completely inside the membrane, its fluorescence response should only show those Cd^2+^ binding to NcHMA4 in its transmembrane domains, not those binding to the rather distant hydrophilic C- and N-terminal tails that have metal binding sites as well (hypothesized to be involved in capturing the metal from the solution). Keeping this in mind, the fluorescence titration results now strongly suggest that HMA4 reaches its maximal ATPase activity when all those internal high-affinity Cd^2+^ binding sites are occupied.

### Expression Analysis of NcHMA4 on the Protein Level

**FIGURE 3:**
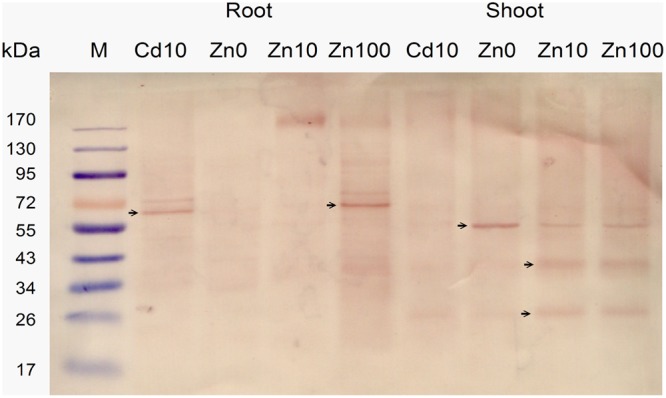
**Analysis of NcHMA4 expression through Western blot with NcHMA4-specific primary antibody (Parameswaran et al., 2007) and Anti-rabbit IgG secondary antibody.** Each lane corresponds to approximately 20 mg of fresh plant tissue from which the proteins were extracted. Three independent replicate plants experiments were carried out, a typical blot is shown.

The expression level of NcHMA4 protein in root and shoot in response to 10 μM Cd and deficient, moderate and high concentrations (“0,” 10, 100 μM) of Zn was analyzed with Western Blots (**Figure 3**). Expression results showed that in roots the well identified 60–70 kD protein was present, at 10 μM Zn replaced by an oligomer of several hundred kD that was also weakly visible at 100 μM Zn. In shoots, along with the 60–70 kD protein, additional low molecular protein bands, strongest around 20 and 40 kD, were detected. These low molecular weight proteins in shoot were often more prominent than the regular high molecular weight NcHMA4, which also appeared slightly smaller than in the roots. In shoots, the low MW versions were expressed in all treatments except the Zn deficient one, which suggests that they may play a role in metal binding, rather than just being degradation products. Therefore, their potential physiological function becomes an interesting question for future work. The 20 kD protein was also observed during purification of NcHMA4 showing the presence of His residues, though it was eluted much before NcHMA4. Characterization of this protein might lead to important information about metal tolerance mechanism in plants. In the roots, the 20 and 40 kD fragments of NcHMA4 were not found in relevant amounts. The regular NcHMA4 (60–70 kD) was more expressed in 10 μM Cd and 100 μM Zn treated plants while in shoots the higher expression was found in Zn deficient and high Zn, i.e., 100 μM treated plants. The results suggest that in roots the NcHMA4 expression was enhanced only in excess of metal (either Cd^2+^ or Zn^2+^).

### Transcription Regulation of *HMA3* and *HMA4* from *N. caerulescens* and *A. halleri* Analyzed on the Organ Level by Real Time PCR (RT-PCR)

**FIGURE 4:**
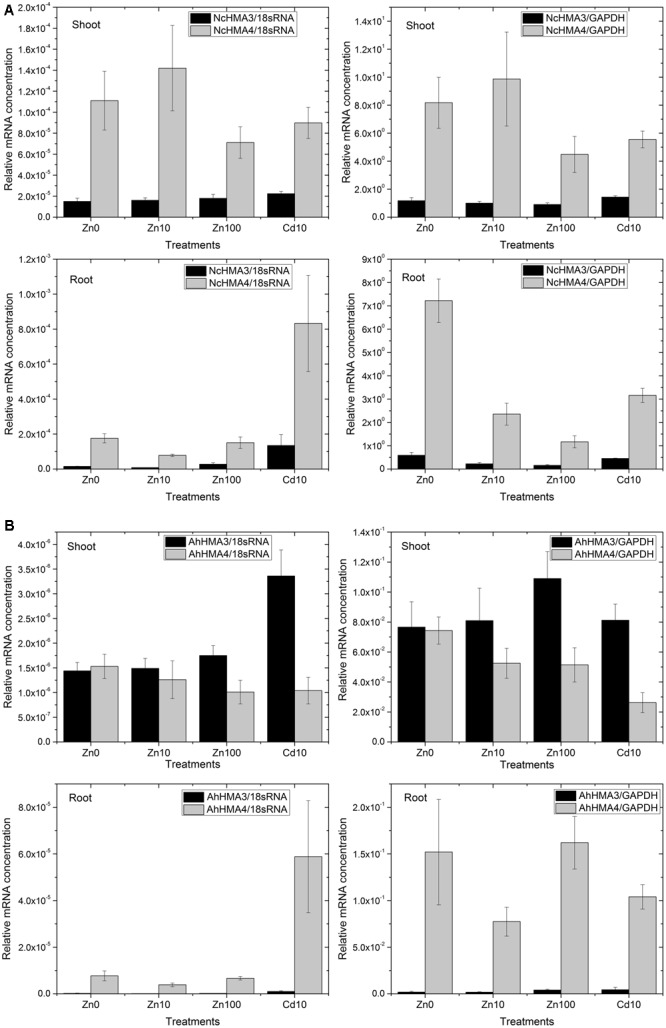
**Differential expression of HMA4 and HMA3 in roots and shoots from *N. caerulescens* (A)** and *A. halleri* **(B)** analyzed by RT – Real time PCR. The data show the mean ± SE from triplicate samples (different plants within the same experiments) and two representative experiments (i.e., six plants total per treatment).

Importantly, in average the abundance of *NcHMA4* mRNA in shoots was similar to the levels in roots (**Figure 4A**). At 10 μM Zn^2+^, *NcHMA4*/18sRNA was at about 0.00009 in roots, while it was at 0.00014 in shoots. At 100 μM Zn^2+^, it was about 0.00015 in roots and 0.00008 in shoots. With normalization to cGAPDH, it was slightly higher in shoots than in roots, but still in the same order of magnitude. This shows that this gene is not only a root-associated metal transporter as reported at the time of its discovery (Papoyan and Kochian, 2004) but also plays role in other physiological functions, which will be a topic for future studies. Papoyan and Kochian (2004) reported very low expression of *NcHMA4* in above ground parts and suggested that it is primarily a root-associated metal transporter. In contrast, in the present study, the expression of *NcHMA4* in shoots was equal, if not higher, to the roots, when judged by these mRNA data as well Western Blots for the analysis of the protein level (**Figure 3**). The differences to earlier studies in shoot levels of *NcHMA4* might be due to the long term of the current study, investigating mature plants instead of seedlings. This is in line with the study of Küpper and Kochian (2010) on cellular regulation of other metal transporter genes, in which the expression of metal transporter genes were different in young and mature plants as well as in the leaves of different developmental stages.

Generally, looking at both roots and shoots and at all metal treatments, transcript levels of *NcHMA4* in *N. caerulescens* were approximately 20× to 100×, respectively, higher than those of *AhHMA4* in *A. halleri* (**Figure 4A** vs. **Figure 4B**). This extreme expression in *N. caerulescens* could be one of the reasons for the well-known fact (reviewed by Leitenmaier and Küpper, 2013) that the Ganges population of *N. caerulescens* has a higher Cd bioaccumulation factor than any other known species. Further, it is one reason why *N. caerulescens* was a better choice than *A. halleri* for the HMA4 protein work using the natural expression.

Furthermore, RT-PCR results normalized to cGAPDH as a reference gene for metabolic activity showed that in roots, *NcHMA4* was significantly up-regulated in response to the Zn deficient condition and toxic Cd^2+^, when compared to the 10 μM Zn^2+^ treatment. This corresponds to the observation by Papoyan and Kochian (2004) of *N. caerulescens* seedlings at the mRNA level (Northern blot). They found higher expression of *NcHMA4* in the roots of plants grown under Zn deficient and high Zn concentrations as well as high Cd concentrations. The current results furthermore show that in response to Zn deficiency and Cd toxicity stress, the increase of *NcHMA4* transcript levels was stronger in roots than in shoots. In shoots, *NcHMA4* mRNA was significantly more abundant in the 0 and 10 μM Zn^2+^ treatments than at 100 μM Zn^2+^ or 10 μM Cd^2+^, but no significant difference existed between the “0” μM and 10 μM Zn^2+^ treatments. *AhHMA4* mRNA was significantly down-regulated in shoots with 10 μM Cd^2+^ treatment (**Figure 4B**), similar to the observation of Mills et al. (2003) where the expression in shoots was down-regulated by exposure of *A. thaliana* to Cd.

Using normalization to 18S-rRNA as an indicator of overall transcription activity, due to higher noise of the data a statistically significant change was recorded only in shoots of *N. caerulescens and A. halleri.* There, *NcHMA4* was up-regulated in the 10 μM Zn^2+^ treatment in comparison with the 100 μM Zn^2+^ treatment.

In contrast to the strong expression of *HMA4* in roots and shoots, in both species *HMA3* was much more expressed in the shoots than in the roots (**Figure 4**). In *A. halleri*, the HMA3 transcript level in roots was even lower than in *N. caerulescens*. Its transcript abundance was rather constant regardless of the Zn^2+^ concentration in the nutrient solution. But compared to both internal standards in both roots and shoots of *N. caerulescens*, as well as compared to 18S-rRNA in *A. halleri*, it was up-regulated in response to 10 μM Cd^2+^. Compared to the transcript abundance in the non-stressed plants, the increase caused by toxic Cd^2+^ was stronger in roots than in shoots (**Figure 4**). Such response to cadmium treatments indicates that in roots, *HMA3* has a function mostly in preventing toxicity.

### Cellular Transcription Regulation of *HMA3* and *HMA4* from *N. caerulescens* and *A. halleri* Analyzed by QISH

**FIGURE 5:**
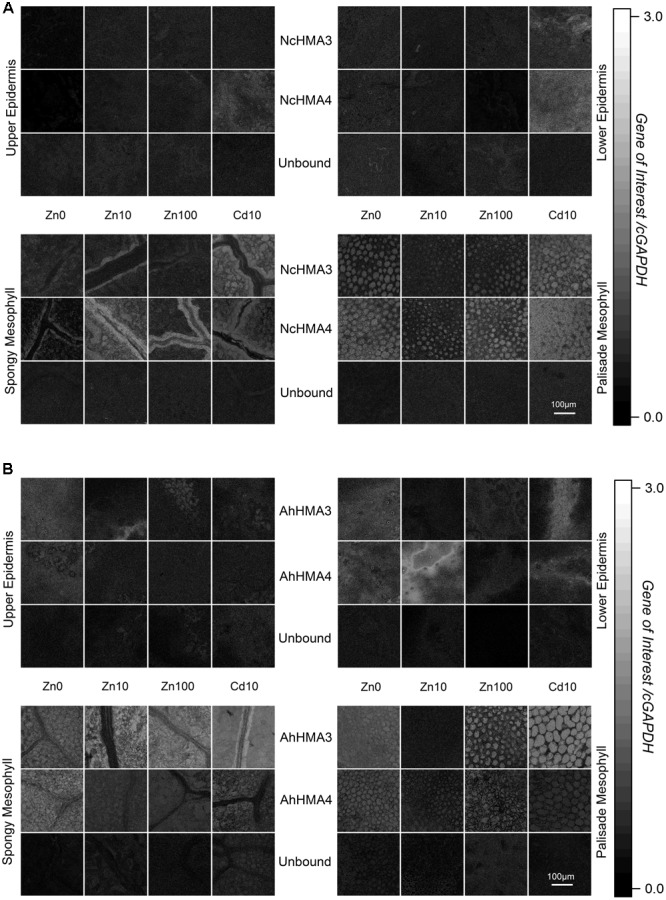
**Cellular expression regulation of HMA3 and HMA4 in *N. caerulescens* (A)** and *A. halleri* **(B)** using QISH technique. Quantitative images showing the background-corrected concentration ratio of the *transcripts* of the genes of interest and cGAPDH probe fluorescence signals in a horizontal optical section through a mature leaf of respective plants grown on metal treatments (Zn^2+^ and toxic Cd^2+^). Scale bar = 100 μm. Autofluorescence images of the same samples that are displayed in this figure are shown in **Supplementary Figure S1** to display the cellular structures of the analyzed tissues. The figure is representative of four independent experiments.

Already the quantitative expression profiles on the organ level (response to metal treatments, locations of expression, and protein processing, as discussed above) of both HMA3 and HMA4 indicated that their roles are more diversified than considered previously. The analysis on the single-cell level by QISH revealed further details how this may be related to the known metal accumulation patterns of *N. caerulescens* and *A. halleri* (**Figure 5** for QISH ratio images; for better structural visibility we also show autofluorescence images of the same samples in **Supplementary Figure S1**). NcHMA4 was already known to be involved in xylem loading/unloading of heavy metal (Papoyan and Kochian, 2004) whereas, HMA3 is involved in vacuolar cadmium sequestration (Morel et al., 2009). Both processes are decisive for hyperaccumulation of these metals, as main differences between hyperaccumulators compared to non-accumulator plants are the enhanced xylem loading and the sequestration of high millimolar concentrations into the vacuoles of (usually epidermal) metal storage cells (review, e.g., by Küpper and Kroneck, 2005; Leitenmaier and Küpper, 2013). Further, the sequences of both genes (cDNA level) are rather different – for example, the *HMA3* genes have less than half the number of cysteines compared to *HMA4* genes. Therefore, the comparison of cellular expression patterns of *HMA3* and *HMA4*, and tissue and metal-dependent transcriptional regulation between *N. caerulescens* (*NcHMA3, NcHMA4*) and *A. halleri* (*AhHMA3, AhHMA4*) is highly interesting.

In *N. caerulescens* both *HMA3* and *HMA4* mRNA levels were highest in the bundle sheath of the vein (**Figure 5A**) while in *A. halleri*, they were highest in the mesophyll (**Figure 5B**). This difference makes sense in terms of the different storage sites of the hyperaccumulated metal (Cd and Zn) in both species. While *N. caerulescens* (like most hyperaccumulators) mainly stores the metal in the epidermal storage cells (Küpper et al., 1999; Leitenmaier and Küpper, 2011), *A. halleri* stores it in the mesophyll (Küpper et al., 2000). Thus it is logical that in *A. halleri* the mesophyll cells have the highest expression of these ATPases (especially for *HMA3*, which is known to be involved in sequestration), while the mesophyll cells of *N. caerulescens* have less *HMA* expression. The transport pathway from the bundle sheath of veins to the epidermal storage cell in *N. caerulescens*, however, remains unknown and an important topic for future research. For both genes, expression in the epidermis was minimal (**Figure 5A**), showing that they are not decisive for the final translocation into the epidermal metal storage cells that are the main metal storage site in *N. caerulescens* (Küpper et al., 1999; Leitenmaier and Küpper, 2011). In these cells, earlier QISH results suggested that *ZNT5* might be responsible for uptake into the cells, and *MTP1* for sequestration to the vacuole (Küpper and Kochian, 2010), both together leading to the much higher metal uptake rates of the epidermal storage cells compared to mesophyll cells (Leitenmaier and Küpper, 2011).

## Conclusion

As key finding on the protein level, metal titration results from the current study compared to our earlier analysis of activity optima (Leitenmaier et al., 2011) show that NcHMA4 reaches its maximal activity only when all high-affinity metal binding sites are occupied – but further addition of metal causes a decrease of activity. The occurrence of a 20 kD fragment of NcHMA4 in shoots at all treatments except the zinc-deficient one suggests a physiological, probably metal-binding, role of this fragment. The expression pattern (response to treatments and cellular differences) of *NcHMA4* and *AhHMA4* revealed that in roots HMA4 plays major role in transport of metal from high metal (Cd or Zn) enriched substrate, while in shoots it is involved in distribution/unloading of metals to storage sites. The investigation of *NcHMA4* expression regulation (both on the protein and the mRNA level) revealed elevated expression at zinc deficiency compared to zinc-replete conditions, which is consistent with the well-known fact that the Cd and Zn bioaccumulation factor in the plants is highest at the lowest Cd (and Zn) concentrations in the soil or nutrient solution. The high expression of *NcHMA4* at toxic Cd and high but not toxic Zn might be playing a role in acclimation to metal toxicity stress, diminishing Cd toxicity and preventing Zn toxicity. Increased expression of *AhHMA3* and *NcHMA3* in roots in response to Cd indicates a role of HMA3 in preventing toxicity. All the expression results (protein and mRNA on the organ level, mRNA at the cellular level) suggest that the role of NcHMA4 is more diversified after long term exposure to metal or in mature plants in comparison to seedlings.

## Author Contributions

SM: Did most of the experimental work and data analysis of the protein biochemistry (including protein expression) part, did the first three experiments for the cellular expression analysis (mRNA level) via QISH, wrote the first draft of the manuscript and participated in later revisions. AM: Did most of the experimental work and data analysis of tissue-level mRNA quantification via qPCR, added the fourth experiment for the cellular expression analysis (mRNA level) via QISH, participated in the writing and revision of the manuscript. HK: Initiated, planned and supervised the project, and received the main funding for it as PI (from the Deutsche Forschungsgemeinschaft and the Ministry of Education, Youth and Sports of the Czechia). Participated in the experimental work, the data analysis, the writing and revision of the manuscript.

## Conflict of Interest Statement

The authors declare that the research was conducted in the absence of any commercial or financial relationships that could be construed as a potential conflict of interest.

## Supplementary Material

FIGURE S1 **Autofluorescence images showing the cellular structures of the *N. caerulescens* (A)** and *A. halleri* **(B)** samples of which the mRNA concentration ratios are shown in **Figure 5**. The figure is representative of four independent experiments.
