# Supplementary figures and images for: Protein Biochemistry and Expression Regulation of Cadmium/Zinc Pumping ATPases in the Hyperaccumulator Plants Arabidopsis halleri and Noccaea caerulescens
Source: Front Plant Sci. 2017 May 22;8:835. doi: 10.3389/fpls.2017.00835 (PMC5438989; doi:10.3389/fpls.2017.00835)

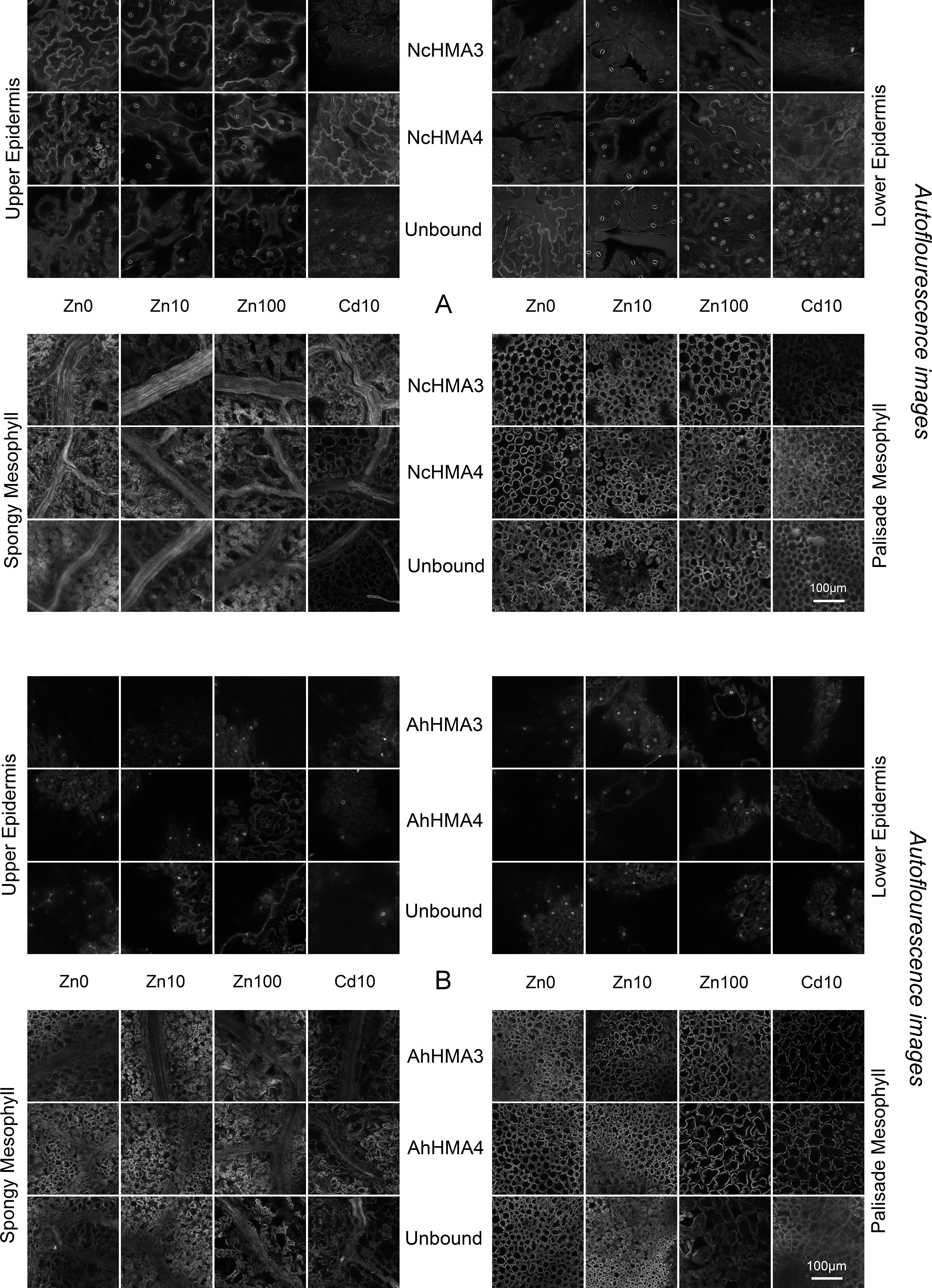

Supplement: FIGURE S1 — Autofluorescence images showing the cellular structures of the N. caerulescens (A) and A. halleri (B) samples of which the mRNA concentration ratios are shown in Figure 5. The figure is representative of four independent experiments. [file Image_1.JPEG]
